# Adenosine and Stroke: Maximizing the Therapeutic Potential of Adenosine as a Prophylactic and Acute Neuroprotectant

**DOI:** 10.2174/157015909789152209

**Published:** 2009-09

**Authors:** Rebecca L Williams-Karnesky, Mary P Stenzel-Poore

**Affiliations:** Department of Molecular Microbiology and Immunology, Oregon Health and Science University, 3181 Sam Jackson Park Road, Portland, OR 97239, USA

**Keywords:** Adenosine, adenosine receptors, cerebral ischemia, neuroprotection, preconditioning, stroke, treatment.

## Abstract

Stroke is a leading cause of morbidity and mortality in the United States. Despite intensive research into the development of treatments that lessen the severity of cerebrovascular injury, no major therapies exist. Though the potential use of adenosine as a neuroprotective agent in the context of stroke has long been realized, there are currently no adenosine-based therapies for the treatment of cerebral ischemia and reperfusion. One of the major obstacles to developing adenosine-based therapies for the treatment of stroke is the prevalence of functional adenosine receptors outside the central nervous system. The activities of peripheral immune and vascular endothelial cells are particularly vulnerable to modulation *via *adenosine receptors. Many of the pathophysiological processes in stroke are a direct result of peripheral immune infiltration into the brain. Ischemic preconditioning, which can be induced by a number of stimuli, has emerged as a promising area of focus in the development of stroke therapeutics. Reprogramming of the brain and immune responses to adenosine signaling may be an underlying principle of tolerance to cerebral ischemia. Insight into the role of adenosine in various preconditioning paradigms may lead to new uses for adenosine as both an acute and prophylactic neuroprotectant.

## INTRODUCTION

Ischemia occurs when blood flow to an organ is inhibited, resulting in a lack of delivery of oxygen and nutrients, and a decreased clearance of potentially toxic metabolic waste products. The brain is selectively vulnerable to ischemia, as it does not store glycogen but instead relies on glucose from the blood. The major strategies used to treat stroke focus on the re-establishment of blood flow to affected tissue. This process of reperfusion has the potential to increase damage to ischemic tissue by generating oxygen-derived free radicals. Thus, it is essential to develop therapies that target both ischemic and reperfusion-related injury in stroke.

Cerebral ischemia is one of the leading causes of morbidity and mortality in the United States. Each year, more than 780,000 individuals suffer from a stroke [94], 87% of which are ischemic [53]. In addition to stroke, brain ischemia and reperfusion (IR) injury can be caused by cardiac arrest. In fact, brain injury affects at least 1/3 of individuals who survive a heart attack [93]. Development of novel drugs for cerebral ischemic injury is a necessity. Many instances of stroke are predictable; as many as 0.7% of *all* surgery patients will suffer from surgery-associated brain ischemia [55], while 10% of patients undergoing cardiac or vascular surgeries will suffer from a frank stroke during or after surgery [11, 15, 40, 55, 69, 76]. Prophylactic neuroprotection has emerged as a promising field of study. Adenosine, an endogenous purine nucleoside that modulates many physiological processes and its receptors have shown great promise as therapeutic targets in the reduction of damage following experimental stroke. In addition to their value as post-stroke treatments that reduce the extent of stroke damage, these compounds are prime candidates for prophylactic neuroprotection.

## BRIEF HISTORY OF ADENOSINE-BASED THERAPIES IN STROKE

For some time adenosine and its receptors have been viewed as potential therapeutic targets for the treatment of stroke. In practice, however, adenosine-based therapies for the treatment of stroke have proven complex in their implementation [107]. Difficulties stem from the widespread distribution of adenosine receptors within the central nervous system (CNS) and throughout the body. For this reason, even direct targeting of specific adenosine receptors leads to widespread off-target effects. Further complications in developing robust adenosine-based therapeutics include imperfect targeting of specific receptor subtypes by agonists and antagonists. Finally, stroke is comprised of a complex set of pathophysiological processes that are influenced differentially by adenosine spatially and temporally.

## BASIC PHYSIOLOGY OF ADENOSINE AND ITS RECEPTORS

The adenosine receptor family consists of four members: A_1_, A_2A_, A_2B_ and A_3_ receptors. Adenosine receptors consist of seven transmembrane spanning regions, and are coupled to G proteins, which activate a number of intracellular signaling pathways [45]. Adenosine receptors are broadly grouped into two categories: A_1_ and A_3_ receptors, which couple to inhibitory G proteins, and A_2A_ and A_2B_ receptors, which couple to stimulatory G proteins. However, adenosine receptors are pleiotropic; they can couple with various G proteins and transduction systems according to their degree of activation and their particular cellular or subcellular location [24]. 

Extracellular adenosine levels are low under normal conditions, but increase substantially in response to metabolic stress [10, 29, 45]. Extracellular adenosine levels are increased under stress *via* several mechanisms: 1) inhibition of equilibrative nucleoside transporters, 2) increased release of ATP from cells and stimulation of extracellular breakdown of ATP by ectonucleotidases, and 3) inhibition of intracellular adenosine removal [10, 29]. Rapid elevations in adenosine occur following ischemia *in vitro* such that within 10 minutes of ischemia adenosine levels in hippocampal slices are increased by more than 20μM [82]. *In vivo* studies also show increases in adenosine in the CNS with cerebral ischemia [61]. *For a more thorough discussion of the source of adenosine in ischemia and in epilepsy, please see Frenguelli & Dale, this issue*. Interestingly, increased levels of adenosine during stroke are not limited to the CNS, as adenosine levels in serum have also been shown to increase following transient ischemic attack (TIA) and stroke in humans [62].

## THE EFFECTS OF ADENOSINE RELEASE ON THE CNS DURING STROKE

Adenosine is a potent endogenous neuromodulator with known neuroprotective properties [23, 37, 91]. Administration of adenosine to the brain at the time of stroke ameliorates damage [57]. Further, transgenic overexpression of adenosine kinase, the primary negative enzymatic regulator of adenosine, leads to increased vulnerability to ischemic cell death, presumably due to decreased levels of adenosine in the CNS [85]. In practice, however, the clinical use of adenosine as a neuroprotectant during stroke has been unsuccessful. Adenosine is an imperfect neuroprotectant, as it can provide benefit in some cases and exacerbate tissue damage in others [23, 84]. Adenosine receptors are distributed throughout the body, and play a prominent role in cardiac and renal system function. When administered intravenously, adenosine at bolus doses of 6-12mg is used to treat patients with paroxysmal supraventricular tachycardia to restore normal sinus rhythm by slowing conduction through the A-V node in the heart [32]. At higher doses, adenosine causes decreased blood pressure by decreasing vascular resistance. Adenosine is also a respiratory stimulant, and intravenous administration can increase ventilation and reduce arterial PCO_2_ causing respiratory alkalosis. Decreased blood flow and respiration compound cerebral ischemic injury, superseding the potential neuroprotective properties of systemically administered adenosine. 

Targeted delivery of adenosine to the CNS in a controlled manner has also proven problematic. Adenosine has a very short half-life in human plasma (<5 seconds), and low blood brain barrier permeability [81]. Molecules with greater plasma stability have been tested such as adenosine analogs, receptor-specific agonists and antagonists, and enzymatic regulators of adenosine. Pretreatment with the unspecified adenosine agonist NECA (adenosine-5prime-N-ethylcarboxamide) protects human astrocytoma cells from apoptosis under hypoxic conditions *in vitro* [8]. Success has also been reported with adenosine agonists in rodent models of cerebral ischemia. Systemic administration of the adenosine kinase inhibitor GP683 at a moderate, but not high or low dose, to rats within 270 minutes of onset of surgical ischemia significantly reduces cerebral damage [104]. 

Unfortunately, the optimal window of administration for adenosine-modulating drugs has been difficult to determine. This is a common problem encountered by many potential stroke therapeutics that show promise in animal models, as accurate determination of the time of stroke onset is difficult in human patients. Stroke patients routinely face delays in access to clinical care, often due to a failure to recognize stroke symptoms. The mean hospital arrival time for patients suffering from stroke is approximately 11 hours after the onset of vessel obstruction [4]. Once patients have been admitted to the hospital, more time may elapse before accurate diagnosis and treatment begins. Thus, stroke drugs with long time windows of efficacy are most desirable. In the case of adenosine, there is the added complication of a diverse distribution of adenosine receptors in the brain, whose actions can be opposing, either spatial or temporally. While administration of a specific adenosine receptor agonist early on may be protective, administration of the same drug late in the course of stroke may exacerbate ischemic damage. 

Direct administration of adenosine-modulating drugs to the CNS is used in animal models to dissect the systemic and CNS effects of adenosine in stroke. These targeted experimental therapies have shown efficacy in reducing ischemic damage in small animal models of stroke. Intracerebral ventricular (ICV) administration of the selective A_3_R agonist Cl-IB-MECA (2-chloro-N6-(3-iodobenzyl) adenosine-5'-N-methylcarboxamide) decreases infarct volume in a mouse model of transient ischemia [18]. Clinically, ICV administration of drugs is impractical and the uncertainty about the diverse temporal effects of adenosine receptor activation and blockade remains problematic. While animal experiments using ICV administration might not translate directly into clinical therapies, they provide insight into the local temporal effects of adenosine on the CNS. The specific functions of the adenosine receptors in the CNS in stroke have been further illuminated by the development of increasingly selective adenosine receptor agonists and antagonists and the creation of mice with targeted deletions in the adenosine receptor system (Table **1**).

### A_1_R Effects in the CNS

In the brain, the A_1_ subgroup is the most abundant and widespread of the adenosine receptors [24]. A_1_ receptors have the highest affinity for adenosine of all the subtypes [29]. Under basal conditions, adenosine likely acts primarily *via* this receptor subtype. Based on immunohistochemical studies, A_1_ receptor expression has been shown to be high in the hippocampus, cerebral cortex, basal ganglia, and some thalamic nuclei in humans and a variety of animals. A_1_ receptor expression is highest in neurons, but the receptor is also present on astrocytes, microglia, and oligodendrocytes [24]. A_1_ receptors are a central focus in the study of the neuroprotective role of adenosine in stroke due to their high concentration and affinity for adenosine, known coupling to inhibitory G proteins and the large number of selective agonists and antagonists available for use. 

Activation of A_1_ receptors is generally considered protective in the context of noxious stimuli. The brain uses vast amounts of ATP generated by oxidative phosphorylation to maintain control of K^+^ and Na^+^ gradients in neurons. Function of the Na^+^/K^+^ ATPase is disrupted during ischemia because mitochondria are unable to make ATP when glucose and oxygen levels are low. Altered Na^+^/K^+^ ATPase activity leads to depolarization of the neuronal plasma membrane and subsequent release of excitatory neurotransmitters. As a result, calcium-permeable NMDA channels open causing calcium-meditated cell death. *For a more complete review of excitotoxic cell death in stroke, please see Doyle K. P. et al., 2008* [28]. A_1_ receptors are linked to inhibitory G proteins, which inhibit adenylyl cyclase, activate inward rectifying K^+^ channels, inhibit Ca^2+^ channels, and activate phospholipase C [10]. This has the cumulative effect of decreasing neuronal excitability. Activation of A_1_ receptors also leads to feedback inhibition of release of presynaptic excitatory neurotransmitters, such as glutamate. Stimulation of A_1_ receptors can therefore lead to prevention of excitotoxic cell death under ischemic conditions.

In practice, A_1_ receptor activation has been shown to produce conflicting effects in both *in vivo *and *in vitro* models of cerebral ischemia. As neurons in the CA1 region of the hippocampus are particularly susceptible to hypoxic cell death, this region is often used as an indicator of tissue damage in both *in vitro* and *in vivo* models of ischemia. In hippocampal slice preparations recovery from hypoxia, as measured by recovery of field EPSP (fEPSP) in the CA1 region, was attenuated by the application of the A_1_R antagonist DPCPX (1,3-dipropyl-8-cyclopentylxanthine) [97]. This result would suggest that antagonists of A_1_R have a detrimental effect in hypoxia, at least at the neuronal level. However, application of the A_1_R antagonist 8-CPT (8-cyclopentyl theophylline) to hippocampal slices at the time of insult does not exacerbate hypoxia induced cell death in the CA1 region of hippocampus [78]. Hippocampal slices from A_1_R knockout mice subjected to hypoxia show a small, though not statistically significant decrease in damage to neurons in the CA1 region [78]. A_1_R knockout mice, when exposed to global ischemia, show no increased neuronal damage in the CA1 region of the hippocampus, the cortex, or the striatum as compared to wildtype controls [78]. Mice treated with 8-CPT at the time of global ischemia have a significant increase in cell death in the CA1 region, the cortex and the striatum when compared to control-treated animals. Taken together, these results suggest that the protective effects of A_1_R activation are temporally regulated. Perhaps acute stimulation *via* A_1_ receptors provides protection by decreasing synaptic transmission, but during recovery ischemia-induced down-regulation of A_1_ receptors may be beneficial.

### A_2A_R Effects in the CNS

A_2A_ receptors are also distributed widely throughout the brain. However, they are expressed at lower density in most regions than A_1_ receptors [24]. The highest density of A_2A_ receptors can be found in the striatum [29]. The affinity of A_2A_ receptors for adenosine is also relatively high. The advent of more selective A_2A_ receptor agonists and antagonists, and the generation of A_2A_ knockout mice, has revealed an important role of A_2A_ receptors as mediators of neuroprotection in stroke. Because they are coupled to excitatory G proteins, it would seem that stimulation of A_2A_ receptors would exacerbate ischemic damage. Paradoxically when applied centrally, A_2A_ receptor antagonists afford protection in several *in vivo* models of ischemic injury [38, 70, 72, 83, 108]. Further, A_2A_ receptor knockout animals show a significant decrease in damage following focal cerebral ischemia [20]. The location of A_2A_ receptors in the brain may also influence ischemic injury. In a model of permanent focal ischemia, a decrease in A_2A_ receptor ligand affinity and an increase in receptor expression were seen in the striatum, but not the cortex [105]. Stroke can occur in diverse regions of the brain depending on the specific vessels involved. It may be possible to tailor adenosine receptor modulating drugs to patients based on the area of ischemia as more is learned about the location specific properties of adenosine receptors. Perhaps A_2A_ receptor antagonism would be a more potent therapy than for patients with striatal strokes than those with primarily cortical injury.

Surprisingly, peripheral stimulation of A_2A_ receptors has also been shown to provide protection to cerebral ischemia *in vivo *[98, 108]. Systemic administration of A_2A_ receptor agonists protect against spinal chord ischemia-reperfusion and traumatic spinal chord injury *in vivo* [17, 89]. Direct administration of A_2A_ receptor agonists to the brain immediately preceding intracerebral hemorrhage reduce cell death [68]. In these cases, protection afforded by A_2A_ receptor agonists is attributed to a reduction of neutrophil infiltration into the affected tissue. Stimulation of A_2A_ receptors has been shown to decrease production of proinflammatory cytokines by peripheral immune cells [14, 64], which may play a role in exacerbating injury in the CNS. However, elucidation of the distinct contributions of A_2A_ receptors in the CNS and in the periphery to ischemic damage has proven difficult. 

A_2A_R knockout bone marrow chimera mice have been created to separately address the peripheral and CNS effects of A_2A_ receptors. As stated previously, A_2A_R knockout mice are protected from cerebral ischemic damage [20]. Similarly, A_2A_R knockout mice are protected from compression injury of the spinal chord when compared to wild-type controls [63]. Chimeric mice lacking A_2A_ receptors in non Bone Marrow Derived Cells (BMDCs) also show protection from compression, but only a small reduction in damage due to infarct is seen in these mice. Notably, protection in spinal compression injury is abolished in wild-type/knockout bone marrow chimera mice that lack the A_2A_ receptor in BMDCs but is retained in cerebral ischemia [20, 63]. Interestingly, the amelioration of damage in chimeric mice lacking A_2A_ receptors on BMDCs is not as robust as in global A_2A_ knockout animals. These results suggest that neuroprotection in these two models of injury may be mediated differentially. In the case of ischemic injury, over 90% of the microglia in the damaged area of chimeric mice were found to be brain derived, indicating that suppression of inflammation in peripheral BMDCs may not have been significant in this circumstance [20]. A_2A_ receptor activation may be beneficial acutely in the periphery by acting on brain endothelium to increase cerebral blood flow and by reducing inflammatory cytokine production by immune cells, while A_2A_ receptor antagonism may be beneficial in the CNS during later stages of ischemia, when prolonged activation of neurons could be detrimental. *For a more thorough discussion of A_2A_ receptors as a target for neuroprotection, please see Roderigo et al., this issue. Additionally, A_2A_ receptor pharmacology is discussed in more detail in the review by Shen et al., also in this issue.*

### A_2B_ Receptor Effects in the CNS

The effects of adenosine A_2B_ receptors during ischemia are not as well characterized as those of A_2A_ receptors. A_2B_ receptors are perhaps the least studied member of the adenosine receptor family. A_2B_ receptors are present on vascular smooth muscle and endothelial cells, as well as on astrocytes [34, 54]. Due to their low affinity for adenosine, and their relative paucity in the brain, A_2B_ receptors seem to be activated only under hypoxic or ischemic conditions, when levels of adenosine surge [59, 92]. Recent *in vitro *studies have suggested a role for A_2B_ receptors in production of glial cell line-derivate neurotrophic factor (GDNF) by astrocytes [114]. Adenovirus-mediated GDNF pretreatment has been shown to reduce cell death following Middle Cerebral Artery Occlusion (MCAO) by both intracerebral and systemic administration [48, 58]. Intracerebral infusion of GDNF post-MCAO increases striatal neurogenesis in rat, indicating a possible role in regeneration as well as protection [60]. A_2B_ receptors may also regulate vascular leak during hypoxia [30], potentially contributing to increased blood flow to affect tissues. Studies of si-RNA mediated A_2B_ receptor suppression *in vitro* have shown increased endothelial leak in response to hypoxia, and A_2B_ receptor knockout animals show increased vascular permeability as compared to wildtype or other adenosine receptor knockout animals [30]. The ablation of the A_2B_ receptor in mice leads to a basal level of low-grade inflammation and increased leukocyte adhesion to the vasculature [115]. A_2B_ receptor mice also display an enhance expression of pro-inflammatory cytokines as compared to control mice. Bone marrow transplantation studies indicate that these processes are regulated primarily by bone marrow, and to a lesser extent vascular, A_2B_ receptors [115]. Taken together, these results indicated that A_2B_ receptors might afford protection to the CNS when activated under ischemic conditions, either directly or by decreasing inflammation and reducing immune cell adhesion to vascular endothelium. 

### A_3_ Receptor Effects in the CNS

A_3_ receptors stimulate inhibitory G proteins. Like A_1_ receptors, A_3_ receptor activation inhibits adenylyl cyclase and leads to the activation of phospholipase C. However, the affinity of A_3_ receptors for adenosine is the lowest of all subgroups, on the order of 6500 nM, which is roughly 100 times lower than the affinity of A_1_ receptors [29]. A_3_ receptors are found throughout the brain, though they are present at levels 10-30 fold lower than A_1_ or A_2_ receptors [107]. Interestingly, A_3_ receptors on CNS cells have been shown to be involved in both cell survival and death, depending on the degree of receptor activation and the specific pathophysiological conditions [1]. More specifically, long duration activation of A_3_ receptors leads to cell death *via* necrosis or apoptosis, while short-term stimulation of central A_3_ receptors prior to injury may reduce spontaneous apoptosis [109]. Application of A_3_ receptor antagonists to hippocampal slices during prolonged oxygen-glucose deprivation (OGD) prevents neuronal loss in the CA1 region [87]. Interesting, during brief periods of OGD, A_3_ receptors antagonism resulted in faster recovery of fEPSP, indicating an inhibitory role of these receptors during initial hypoxia [87]. Chronic pre-exposure to A_3_ agonist is protective *in vivo*; this may be due to rapid desensitization of A_3_ receptors [80, 106]. Intravenous or ICV administration of the selective A_3_ receptor agonist Cl-IB-MECA 165 minutes and 15 minutes prior to the onset of transient ischemia is protective in rats [18]. Consistent with these results, A_3_ knockout animals have increased susceptibility to transient cerebral ischemic injury [18]. Post ischemic administration of Cl-IB-MECA is also protective *in vivo* [110]. Interestingly, in contrast to *in vitro* results, a single dose of Cl-IB-MECA given acutely during focal ischemia increased infarct volume [109]. These results suggest that the effects of A_3_ receptor signaling during ischemic injury may be beneficial or detrimental depending on the specific circumstances in which the stimulus occurs. Correctly timed, A_3_ receptor modulation may be a potential target for therapeutic intervention. Early chronic stimulation of A_3_ receptors leading to their down regulation, or acute A_3_ receptor blockade, may be one strategy for inducing prophylactic neuroprotection in advance of predicted ischemic injury. 

## PAST COMPLICATIONS IN DEVELOPING ADENOSINE-BASED THERAPIES FOR STROKE

A major stumbling block in the development of adenosine-based neuroprotective strategies has been the dramatic systemic effects of adenosine which include decreased heart rate, blood pressure and body temperature, and sedation [9]. As more specific adenosine receptor agonists and antagonists are developed, drugs with fewer off-target effects may emerge. Novel therapeutic methods may also allow for easier local delivery of adenosine, or modulators of specific adenosine receptors, to the central nervous system. *A novel example of one such strategy, the implantation of adenosine-releasing silk polymer to treat epilepsy, can be found in Wilz, A.* *et al.*, 2008 [112]. Additionally, the systemic effects of adenosine may be harnessed to ameliorate damage caused by ischemic stroke. 

## PERIPHERAL IMMUNE INVOLVEMENT AND INFLAMMATORY CYTOKINES IN STROKE

**Fig. (1):**
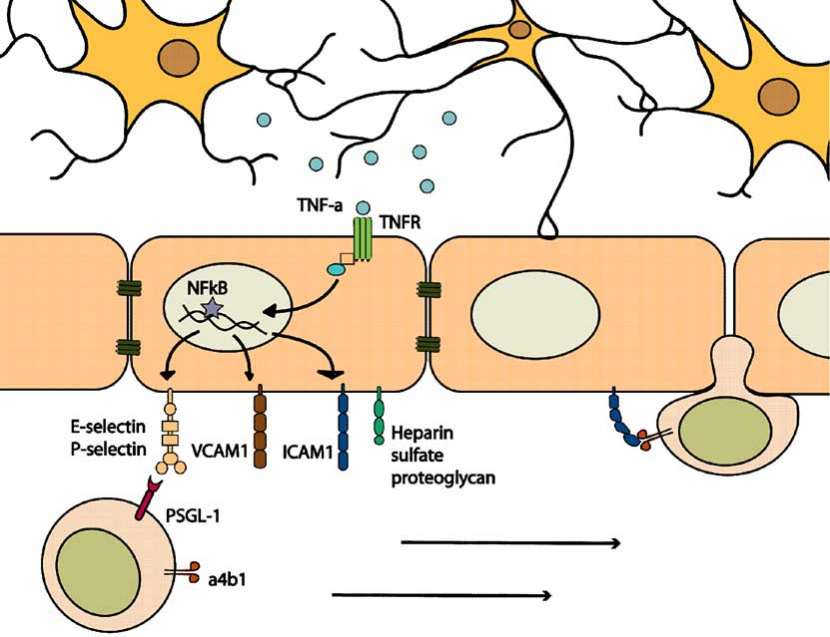
**Peripheral immune involvement following ischemic brain injury.** Neurons and astrocytes damaged by ischemia release pro-inflammatory factors such as TNF-α into the brain parenchyma. These pro-inflammatory molecules in turn diffuse and bind receptors on vascular endothelial cells. Activation of endothelial cells *via* TNF and related receptors leads to nuclear factor kappa B (NFκB) activation and nuclear translocation. In the nucleus, NFκB activity induces transcription of adhesion molecules and chemokines, including E-selectin, P-selectin, vascular cell adhesion molecule (VCAM1), intercellular adhesion molecule (ICAM1) and heparin sulfate proteoglycan. Peripheral immune cells traveling through the vasculature express receptors to adhesion molecules (i.e. PSGL-1 for selectins, α4-βintegrin for VCAM1). As they survey the body, leukocytes roll along the activated endothelium, adhere *via* adhesion molecules and become activated themselves, then extravasate into the brain parenchyma.

In the context of a pathogen invasion, a vigorous inflammatory response is an effective means to clear an invasive microorganism. Responding immune cells kills not only pathogens, but also the cells harboring them. However, when this same response is co-opted by the host to clear infarcted tissue, it can damage the very cells it is intending to save. Much of the damage associated with brain ischemia is due to the resultant inflammatory response. This has been demonstrated by the amelioration of ischemic damage through the use of anti-inflammatory strategies [42, 90, 99]. Inflammation following stroke originates with microglia and astrocytes, which detect injury-associated molecules and produce pro-inflammatory cytokines [31, 46]. Vascular endothelium becomes activated, signaling to the periphery and increasing blood brain barrier permeability [2, 6]. The inflammatory response is further promoted by the infiltration of macrophages and neutrophils (Fig. **1**). These cells produce proteolytic enzymes, inflammatory cytokines and other cytotoxic molecules.

Proinflammatory cytokines have been implicated in exacerbating the damage in stroke by increasing neuroinflammation, with particular attention paid to TNF-α [5]. TNF-α is pleiotropic inflammatory cytokine that is produced by a variety of cell types, including peripheral immune cells and cells that are resident in the CNS. Intravenous administration of anti-TNF-α antibody to mice following transient cerebral ischemia has been shown to ameliorate damage and reduce edema [51]. Adenosine receptor agonists and antagonists are both effective suppressors of TNF-α through their actions on various cell types. In rabbits undergoing transient spinal chord ischemia, lower levels of plasma TNF-α and decreased damage are seen following intravenous administration of the adenosine A_2A_ analogue ATL-146e (4-(3-[6-amino-9-(5-ethylcarbamoyl-3,4-dihydroxy-tetrahydro-furan-2-yl)-9H-purin-2-yl]-prop-2-ynyl)-cyclohexanecarboxylic acid methyl ester) [16]. Understanding how adenosine modulates the release of TNF-α by both peripheral immune cells and by cells in the CNS may be particularly beneficial when developing adenosine based therapies for the treatment of stroke. The effects of TNF-α on adenosine receptor expression may also provide insight into the phenomenon of ischemic preconditioning, a phenomenon in which a brief noxious stimuli induce changes in the CNS and in the periphery that result in endogenous neuroprotection from stroke.

## THE EFFECTS OF ADENOSINE ON THE PERIPHERAL IMMUNE SYSTEM

**Fig. (2):**
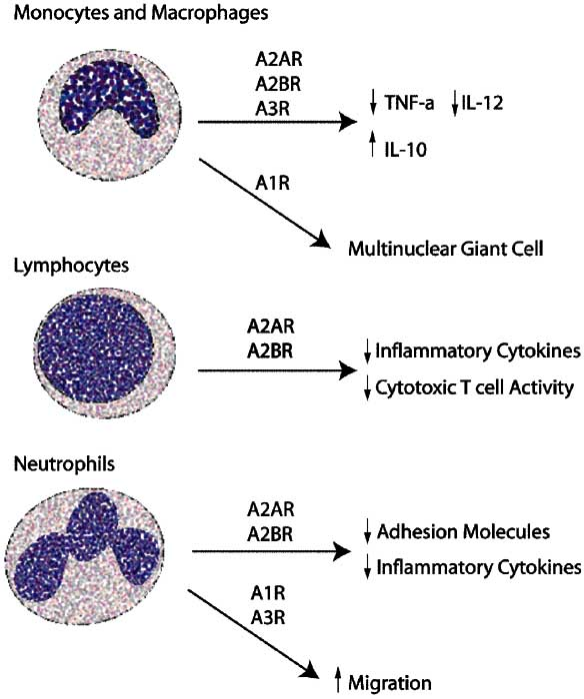
**Adenosine modulates peripheral immune cell function.** Adenosine modulates the activity of peripheral immune cells differentially depending on the receptors that are activated. Overall, activation of A_2A_ and A_2B_ receptors directs immune cells to an anti-inflammatory response. This property could be harnessed in stroke to reduce inflammatory damage in the CNS caused by infiltrating activated immune cells. Stimulation *via* A_1_ receptors on immune cells tends to increase migration and activity, but also leads to angiogenesis through their interaction with cerebral vascular endothelial cells.

Adenosine may play a role in modulating systemic immune involvement in stroke that is in addition to its role in the CNS. Adenosine levels in serum increase following stroke [62]. This finding is particularly significant, as peripheral immune cells express functional adenosine receptors, and respond to changes in extracellular adenosine (Fig. **2**). It is also important to consider the peripheral effects of adenosine-based therapeutics, as systemic administration of adenosine-modulating drugs affect immune cells as well as the CNS.

### Effects of Adenosine on Monocytes and Macrophages

Monocytes and macrophages express the full complement of A_1_, A_2A_, A_2B_, and A_3_ receptors. Stimulation *via* adenosine receptors not only shapes macrophage function, but can also influence differentiation. High levels of extracellular adenosine can delay monocyte maturation [35]. Broadly, extracellular adenosine has the affect of changing the response of macrophages from proinflammatory to anti-inflammatory. Adenosine inhibits TNF-α and IL-12 production, while increasing IL-10 production by macrophages [44]. These effects appear to be primarily mediated *via* A_2A_, A_2B_ and A_3_ receptors [43]. Recent results have shown that A_2B_ receptor activation with the selective agonist BAY 60-6583 on macrophages *in vitro* and *in vivo* leads to decreased production of TNF-α following injurious stimuli [19]. However, it should be noted that adenosine has differential effects on macrophage development depending on the receptor it stimulates. Stimulation *via* A_1_ receptors has been found to influence monocytes to become phagocytic multinuclear giant cells, while stimulation *via* A_2A_ and A_2B_ receptors has been shown to inhibit this process [71]. Monocytes also affect resolution of ischemic damage by influencing vasacular endothelial cells. Application of the selective A_1_ receptor agonist CPA (N^6^-cyclopentyladenosine) to macrophages results in increased production of vascular endothelial growth factor (VEGF), leading to an increase in capillary formation *in vitro* [22]. When activated, macrophages can themselves be a source of extracellular adenosine, *via* ATP production [44]. Through this local extracellular production of adenosine, macrophages may also suppress the inflammatory response of other immune cells. Monocytes and macrophages exert broad influence on damage and healing following stroke. Stimulation of the A_2A_ receptor inhibits production of TNF-α, decreasing inflammation at the time of ischemia. Therapies that increase the expression of A_2A_ receptors, such as the Toll-like receptor 4 (TLR4) agonist lipopolysaccharide (LPS) [102], may be useful as a prophylactic to modulate the response of macrophages to endogenous adenosine release during ischemic injury. 

### Effects of Adenosine on T and B Lymphocytes

Recruitment of activated lymphocytes to the brain parenchyma following stroke leads to increased tissue damage [52]. Adenosine may serve as a negative regulator of lymphocyte activation, and thereby reduce damage caused by an excessive inflammatory response. Lymphocytes express A_2A_, A_2B_ and A_3_ adenosine receptor subtypes [39]. Similar to what is seen in macrophages, stimulation of adenosine receptors on lymphocytes has the net effect of suppressing activation. The adenosine A_2A_ receptor has been directly implicated in adenosine-mediated suppression of cytokine production and cytotoxic activity in natural killer T cells, a highly specialized group of T lymphocytes [50]. Additionally, evidence is mounting to support the hypothesis that adenosine mediates the formation of T regulatory cells [43, 118]. Unlike cytotoxic T lymphocytes, regulatory T cells serve as suppressors of inflammation, and can limit damage to healthy tissues caused by overactivated immune cells. Surprisingly, the regulatory function of T regulatory cells has been linked to their ability to produce adenosine [25]. A_2A_ receptors appear to be the dominant receptor subtype mediating the immunosuppressive effects of adenosine on lymphocytes. Stimulation of the A_2A_ receptor in mixed lymphocyte populations leads to a decrease in expression of the intercellular adhesion molecule ICAM, decreased production of several proinflammatory cytokines, and decreased proliferation [103]. However, it should be noted that the actions of A_2A_ receptor stimulation could be blocked *via* A_1_ or A_3_ receptor activation [103]. While this may be relevant with acute systemic administration of adenosine-based therapeutics selective for A_1_ and A_3_ agonists, the negative consequences could potentially be overcome by using short-acting, CNS-targeted drugs. 

### Effects of Adenosine on Neutrophils and other Granulocytes 

Neutrophils are granulocytes, members of the polymorphonuclear family of cells, which also includes eosinophils and basophils. Following activation by cytokines and chemokines, neutrophils upregulate intracellular adhesion molecules, adhere to vascular endothelium and migrate into damaged tissue. Migration of neutrophils occurs well before migration of monocytes or lymphocytes [113]. Once at the site of inflammation, neutrophils release preformed granule constituents, lytic enzymes, reactive oxygen species, and inflammatory cytokines such as TNF-α [47]. While helpful in the context of response to infection with a pathogen, as with other immune cells, over-activation of neutrophils at sites of injury can exacerbate damage by damaging healthy tissue. Neutrophils in particular are strongly recruited following ischemic-reperfusion injury, and have been shown to contribute significantly to subsequent neuroinflammatory damage [111]. It has long been known that adenosine promotes migration of neutrophils, and it has recently come to light that this occurs *via* stimulation of A_1_ and A_3_ receptors [21, 95]. Conversely, by acting *via* A_2A_ receptors, adenosine down regulates surface expression of adhesion molecules, and decreases the production of pro-inflammatory cytokines [43]. Application of the adenosine kinase inhibitor GP515 attenuates neutrophil degranulation *via* an adenosine-mediated action [12]. This action appears to be mediated strongly by A_3_ receptors, as well as by A_2A_ receptors, but not by A_1_ receptors [12]. In mast cells, A_2A_ receptor stimulation also bocks degranulation, while A_2B_ receptor activation causes release of VEGF [22]. Taken together, these results indicate that A_2A_ receptors serve as a negative regulator of granulocyte-induced inflammation, while A_1_ receptors may promote the inflammatory properties of these cells. A_3_ receptors appear to have mixed effects on neutrophils, depending on the context in which the stimulation occurs. 

Neutrophils infiltrate into the brain parenchyma following ischemic injury, contributing to immune mediated inflammatory damage following stroke; adenosine receptors provide an accessible, potential target for modulation of the proinflammatory properties of these cells. Overall, A_2A_ receptor stimulation on neutrophils acutely and immediately following stroke likely decreases inflammatory damage. Compounds that directly induce expression of A_2A_ receptors on neutrophils such as LPS, or those that elicit the release of cytokines like TNF-α, Interleukin 1β (IL-1β) [36], may be useful as prophylactic neuroprotectants by reprogramming the response of neutrophils to stroke.

## A PUTATIVE ROLE FOR ADENOSINE IN PROPHYLACTIC NEUROPROTECTION

It is well known that certain antecedent treatments can protect patients from damage due to an ischemic episode. Cerebral ischemic preconditioning has emerged as a promising field of study for the development of prophylactic neuroprotectants. Ischemic preconditioning can be induced by a variety of noxious stimuli, including short periods of hypoxia or ischemia, cortical spreading depression, brief seizure, exposure to inhaled anesthetic or low dose of bacterial endotoxin [27]. Preconditioning that occurs rapidly, immediately following stimulation, is termed acute preconditioning. Local increase of adenosine in the brain is likely one of the mechanisms by which acute preconditioning occurs [24]. Rapid upregulation of neuroprotective A_1_ receptors in the brain has been shown to be one mechanism of adenosine-mediated acute preconditioning. When given during a brief conditioning period of ischemia, the selective A_1_ receptor agonist DPCPX attenuates the protective effects of acute preconditioning [3, 75]. Rapid tolerance against focal ischemia by isoflourane is also attenuated by DPCPX administration [65]. As an additional mechanism, rapid adenosine receptor desensitization may contribute to acute neuroprotective as is seen following chronic A_3_ receptor agonism [80]. 

Neuroprotection that develops over a period of several hours or days and requires new protein synthesis is termed delayed preconditioning. As in acute preconditioning, adenosine may play a role in delayed preconditioning. ATP diphosphohydrolase and 5’-nucleotidase, two enzymes involved in the local production of adenosine from ATP *via* hydrolysis, show enhanced activity in the CA1 region of the hippocampus following brief ischemia. This increase in enzymatic activity correlates with protection in the CA1 region from subsequent ischemic injury, given 48 hours later. Adenosine receptor blockade blocks tolerance to ischemia induced by preconditioning. As with acute preconditioning, the neuroprotective effects of ischemic preconditioning are attenuated with the administration of the selective A_1_ agonist DPCPX at the time of conditioning ischemia [49, 116]. Interestingly, DPCPX administered at the time of ischemia, 72 hours after preconditioning, does not reverse protection, implying a role for A_1_ receptors during conditioning ischemia [116]. 

Unmethylated cytosine-guanine rich oligodinucleutides (CpG ODNs), which act on Toll-like receptor 9 (TLR9), have been shown to be a highly effective means of prophylactic neuroprotection in a mouse model of cerebral ischemia [101]. The delayed neuroprotection afforded by CpG ODNs occurs following systemic injection. CpG ODNs also confer protection from oxygen-glucose deprivation in mixed cortical cultures *in vitro*. Similar finding have been previously reported for the TLR4 agonist LPS [96]. An increase in systemic levels of TNF-α is required for neuroprotection in both LPS- and CpG-mediated preconditioning [96, 101]. Interestingly, proinflammatory cytokines such as TNF-α increase the expression and signaling of A_1_ receptors in the brain [7]. Upregulation of A_1_ receptors in the CNS, effectively preparing the brain for ischemic insult, may be one mechanism by which TLR agonists elicit endogenous neuroprotection. TNF-α also upregulates A_2A_ and A_2B_ receptor expression on endothelial cells, which may contribute to suppression of inflammation following ischemic injury [73, 100]. This contribution may be highly relevant, as stimulation of these receptors on vascular endothelial cells suppresses expression of adhesion molecules such as E-selectin and vascular cell adhesion molecule (VCAM-1) [13]. Stimulation of excitatory A_2A_ receptors enhances nitric oxide release and vasodilation [86], and stimulation of A_2B_ receptors leads to expression of angiogenic factors [33]. TNF-α also leads to the upregulation of A_2A _receptors on peripheral immune cells [56]. Upregulation of A_2A_ receptors on neutrophils is seen following stimulation with TNF-α, which may reduce the damage caused by inflammatory immune infiltrate into the brain following stroke [36]. With these specific adenosine receptors increased, immune cells may be more likely to produce an anti-inflammatory response in the setting of stroke especially when in conjuction with increased production of adenosine. In support of this idea, A_2A_ receptors have been shown to decrease TNF-α production and adhesion molecule expression following stimulation with TLR4 agonists [41], which are also released endogenously following stroke [67]. Indeed, synergism between adenosine and TLRs are well documented, especially in peripheral immune cells [66, 77, 88]. While the involvement of adenosine receptors in TLR-mediated preconditioning has not been confirmed, direct regulation of adenosine-related gene products is also seen *via* microarray analysis in both brain and blood following TLR-mediated preconditioning (Stenzel-Poore lab, unpublished observation).* For a more extensive discussion of TLR signaling in endogenous neuroprotection and stroke, please see the 2009 review by Marsh, B. J. et al. [67]. *A greater understanding of the role of adenosine in endogenous neuroprotection may lead to interventions and therapeutic strategies useful in ischemia. A focus on the interaction between TLRs and adenosine receptors in preconditioning may be appropriate because systemic TNF-α levels may remain lower—a feature that is preferable in the treatment of patients.

## CONCLUSION AND PERSPECTIVE

Stroke is a leading cause of death and disability in the United States [74]. Despite years of study, there is only one clinical therapy for the treatment of stroke--removal of thrombus mechanically or *via* lysis by administration of tissue plasminogen activator (tPA) [79]—and tPA is, at best, an imperfect treatment. Thrombolysis is only applicable in the setting of occlusive stroke, treatment must be applied within a short time window, and the risk for hemorrhagic episodes following tPA are high [26]. Though no adenosine-based therapies for treatment of stroke exist, selective activation and inhibition of adenosine receptors in the CNS, especially when combined with peripheral immune modulation, is a promising target for future stroke therapies. Perhaps the most benefit will be gained through the use of prophylactic therapies that alter the expression of adenosine receptors on specific cells types. Utilization of complementary molecules, such as prophylactic TLR-agonists, will also improve stroke treatment by enhancing the benefits of adenosine receptor stimulation. The future success of stroke therapy does not rest on the discovery of a novel miracle compound, but on the intelligent design of multimodal, synergistic treatments that target several sources of damage. 

## Figures and Tables

**Table 1:** Effects of Adenosine on Cerebral Ischemia: Studies with Transgenic Animals

Target	Manipulation	Model	Effect	Reference
Adenosine kinase	Overexpression in brain	tMCAO^a^	3-fold increase in infarct volume vs. wildtype controls^b^	[85]
A_1_R	Global Knockout	Global Ischemia	No change in percentage dead neurons vs. wildtype controls	[78]
A_2A_R	Global Knockout	tMCAO	Reduction in infarct volume vs. wildtype controls	[20]
A_2A_R	Bone Marrow Chimera	tMCAO	WT BMDCs→KO: ~20% decrease in infarct volume, not statistically significant^c^ KO BMDCs→WT: ~30% reduction in infarct volume, improved neurological outcome KO BMDCs→KO: ~60% decrease in infarct volume	[117]
A_3_R	Global Knockout	tMCA ligation	Increased infarct volume vs. wildtype controls	[18]

a tMCAO: transient middle cerebral artery occlusion

b MCAO given for 15 minutes. 60 minute occlusion was lethal in ADK overexpressing animals.

c All animals versus WT BMDCs→WT.
